# Regulation of Gamma-Aminobutyric Acid Transaminase Expression and Its Clinical Significance in Hepatocellular Carcinoma

**DOI:** 10.3389/fonc.2022.879810

**Published:** 2022-06-24

**Authors:** Xiaoqiang Gao, Xiaodong Jia, Moyan Xu, Jiao Xiang, Jin Lei, Yinyin Li, Yinying Lu, Shi Zuo

**Affiliations:** ^1^ Department of Hepatobiliary Surgery, Affiliated Hospital of Guizhou Medical University, Guiyang, China; ^2^ Department of Liver Disease, Fifth Medical Center of Chinese People's Liberation Army General Hospital, Beijing, China; ^3^ Health Care Office, Chinese People's Liberation Army General Hospital, Beijing, China; ^4^ Education Department, Beijing Anzhen Hospital, Capital Medical University, Beijing, China; ^5^ Center for Synthetic and Systems Biology (CSSB), Tsinghua University, Beijing, China; ^6^ Guangdong Key Laboratory of Epigenetics, College of Life Sciences and Oceanography, Shenzhen University, Shenzhen, China

**Keywords:** gamma-aminobutyric acid transaminase, hepatocellular carcinoma (HCC), DNA methylation, copy number variation - CNV, gene mutation, miR-135a-5p, glycolysis (Warburg effect), immune infiltration

## Abstract

**Background:**

Gamma-aminobutyric acid transaminase (ABAT) catalyzes the conversion of gamma-aminobutyric acid (GABA) into succinic semialdehyde. Although some evidence supports a key role of ABAT in the progression of hepatocellular carcinoma (HCC), no systematic analysis is available. Thus, this study aimed to investigate the possible mechanisms related to low ABAT expression and the prognostic value and potential functions of ABAT in HCC.

**Methods:**

We obtained relevant datasets from the Encyclopedia of RNA Interactomes, MethSurv, cBioPortal, TISIDB and The Cancer Genome Atlas and used bioinformatic methods to analyze DNA methylation, copy number variation, gene mutation, and upstream microRNAs (miRNAs) of ABAT, exploring the potential relationship between ABAT expression and the prognosis, glycolysis, and immune infiltration in HCC.

**Results:**

The results indicated that ABAT expression was lower in HCC tumor tissues than in normal tissues or adjacent tissues. Low ABAT expression was related to patient age, T stage classification, pathologic stage, histological grade, and alpha-fetoprotein level of HCC. Kaplan-Meier survival analyses indicated that low ABAT expression was correlated with poor HCC prognosis. ABAT was also verified as an independent risk factor in HCC *via* Cox multivariate analysis. Gene set enrichment analysis showed enrichment in various signaling pathways. Furthermore, DNA methylation, copy number variation, and gene mutation potentially induced low ABAT expression; miR-135a-5p was a potential upstream miRNA of ABAT. Additionally, ABAT expression was associated with glycolysis-related genes, infiltrated immune cells, immunoinhibitors, and immunostimulators in HCC.

**Conclusions:**

Our study reveals that deficient ABAT expression is correlated with disease progression and poor prognosis in HCC because of its role in tumorigenesis and tumor immunity.

## Introduction

Hepatocellular carcinoma (HCC) is a type of primary liver cancer accounting for more than 90% of primary liver cancer cases. The etiological factors of HCC include hepatitis virus infection, alcohol consumption, and nonalcoholic fatty liver disease (1). Despite multiple treatment modalities, the five-year survival rate for HCC remains below 20% worldwide. The failure of HCC screening resulting in late diagnosis, delayed treatment, and underuse of treatment methods in clinical practice may primarily cause the poor prognosis of HCC (2). Accordingly, accurate biomarkers are required for improved early-stage screening and diagnosis of HCC and for improving patient outcomes.

Gamma-aminobutyric acid (GABA) is an important neurotransmitter in the nervous system. The ionotropic GABA-A receptor (GABAA) and the metabotropic GABA-B receptor (GABAB) mediate the biological effects of GABA. GABA is involved in the growth and progression of tumors. Gamma-aminobutyric acid transaminase (ABAT) catalyzes the conversion of GABA into succinic semialdehyde and L-glutamate, and is expressed at low levels in basal-like breast cancer (BLBC), leading to an increase in GABA production. GABA then binds to the GABA-A receptor to increase the concentration of intracellular Ca^2+^, promoting tumor progression by activating nuclear factor of activated T cells 1 (NFAT1) in BLBC cells (3). ABAT is still poorly expressed in HCC, which is correlated with a bad prognosis in patients with HCC, and the proliferation, migration, and invasion of HCC cells are inhibited by the overexpression of ABAT (4). Inconsistent with the study in BLBC (3), the low expression of ABAT may promote HCC progression through other signaling pathways than the GABA pathway. Firstly, GABA-A receptor activation induces the hyperpolarization of hepatic cells, which inhibits hepatic regeneration (5). Secondly, Baclofen, a GABAB receptor agonist, suppresses HCC cell growth by inducing G0/G1 phase arrest (6). Furthermore, GABA suppresses HCC cell migration and invasion through induction of the GABA-A receptor (7). The causes of the low expression of ABAT also remain obscure.

In the present study, we explored the correlation between ABAT and HCC using The Cancer Genome Atlas (TCGA) database. Gene set enrichment analysis (GSEA) was performed to elucidate the ABAT-related biological pathways in HCC. We analyzed DNA methylation, copy number variation, and gene mutations of *ABAT* using the MethSurv and cBioPortal databases. Upstream microRNAs (miRNAs) of ABAT were identified using the Encyclopedia of RNA Interactomes (ENCORI) and TCGA databases, and the relationship between ABAT and glycolysis was analyzed. The correlation of ABAT expression with immune cell infiltration and immunomodulatory molecules was investigated using the TISIDB and TCGA databases.

## Materials and Methods

### ABAT Gene Expression Analysis With TCGA Database

HCC mRNA expression data were obtained from TCGA database (https://genome-cancer.ucsc.edu/). The R package “limma” was used to explore differential ABAT expression by analyzing the data after normalization. The R package “ggplot2” was utilized to draw plots.

### Sample Collection

Tissue samples from 71 HCC patients who underwent surgical resection between 2013 and 2015 at the Department of Hepatic Surgery, in the Fifth Medical Center of the Chinese PLA General Hospital, Beijing, China, were collected. No anticancer treatments were administered to the patients before surgery. The follow-up endpoint for all patients was May 2020.

### RNA Extraction and Quantitative Reverse Transcription Polymerase Chain Reaction

The total RNA content was extracted from the tumor tissues and adjacent non-tumor tissues (at least 2 cm away from the edge of the tumor) using the TRIzol method. Complementary DNA (cDNA) was synthesized based on the manufacturer’s protocol (DBI Bioscience, Newark, DE, USA). qRT-PCR (Thermo Fisher Scientific, USA) was performed with cDNA and qPCR SuperMix (Takara Bio, Inc., Otsu, Japan). Each sample was tested in triplicate, and all samples were tested thrice. The specific primers of ABAT were as follows: 5`-AAGAGAGCCGAGGCAATTACC-3` and 5`-GCTCGCATTTTGAGGCTGTTG-3`. The GAPDH-specific primers were as follows: 5`-GGAGCGAGATCCCTCCAAAAT-3` and 5`-GGCTGTTGTCATACTTCTCATGG-3`. The forward primer of miR-135a-5p (poly (A)−tailing method) was as follows: 5`-TATGGCTTTTTATTCCTATGTGA-3`. Normalization of the results was done using the 2^-ΔΔCt^ value.

### Western Blot

Eight pairs of HCC tissue samples were dissolved using radioimmunoprecipitation assay buffer (BIOSS, Beijing, China). A bicinchoninic acid protein assay kit (BIOSS, Beijing, China) was used to determine protein concentrations. Equivalent protein samples were separated using 12% sodium dodecyl sulfate-polyacrylamide gel electrophoresis and diverted onto a polyvinylidene fluoride membrane (Millipore, USA). The membrane was incubated with primary rabbit anti-human ABAT antibody (1:2000 dilution; Abcam, USA) overnight at 4°C after blocking, followed by Horseradish Peroxidase (HRP)-conjugated goat anti-rabbit secondary antibody (1:4000 dilution; Abcam, USA) for 2 h. Glyceraldehyde-3-phosphate dehydrogenase was used as the control. In the end, the membrane was rinsed with phosphate-buffered saline with 0.1% Tween 20 buffer thrice and scanned with a gel imaging system (Thermo, Waltham, USA).

### Immunohistochemistry

Six pairs of paraffin-embedded samples were incubated with normal goat serum after rehydration and oxidation with hydrogen peroxide. Slides were incubated with primary rabbit anti-human ABAT antibody (1:100 dilution; Abcam, USA) overnight at 4°C followed by HRP-conjugated secondary antibody (Zhongshan Golden Bridge Biotechnology, China) for 30 min. Finally, the slides were sealed with resinene after incubation with 3,3-diaminobenzidine and counterstained with hematoxylin. Five fields were randomly selected and imaged with a microscope (Olympus, Japan) at 200× magnification. Image Pro Plus software (version 7.0; USA) was utilized to analyze the results by calculating the average ratios of integrated optical density (IOD) to the positive area (IOD/pixel).

### Relationships Between ABAT Expression, Clinical Phenotype, and Prognosis

Relevant data were extracted from each sample obtained from TCGA database. Ten clinical phenotypes were selected, namely, patient age, patient gender, T stage classification, N stage classification, M stage classification, pathologic stage, histologic grade, alpha-fetoprotein (AFP) level (ng/mL), Child-Pugh grade, and vascular invasion. The relationship between these clinical phenotypes and ABAT expression was explored. Correlation analysis was conducted using the R packages “limma” and “ggplot2.” Overall survival (OS) was employed to explore the correlation between ABAT expression and patient prognosis. Survival analysis was carried out with the Kaplan-Meier method and log-rank test. The R packages “survival” and “survminer” were utilized to draw the curves.

### Cox Regression Analysis

Logistic regression analysis was performed using the R packages “survival” and “forestplot” to further determine the effect of ABAT expression in HCC patients. A nomogram that predicted the 3- and 5-year survival probabilities based on the Cox regression analysis was drawn using the R package “rms.” The C-index and calibration plot to assess the quality of the nomogram model were obtained using the R package “Hmisc.” The C-index, calculated using a bootstrap method with 1000 resamples, was used to assess the discrimination of the nomogram.

### GSEA

The R package “DESeq2” was utilized to identify the differentially expressed genes (DEGs) by comparing the low- and high- expression data of ABAT in HCC samples of TCGA database (HTseq-Count). The functional and pathway differences were determined by GSEA with the R package “ClusterProfiler.” Adjusted P < 0.05 and false discovery rate (FDR) < 0.25 were deemed statistically significant.

### Correlations of ABAT Expression With DNA Methylation, Copy Number Variation, and Gene Mutation

Gene expression is affected by many factors. To explore the aspects that affected the expression of ABAT, the MethSurv (https://biit.cs.ut.ee/methsurv/) and cBioPortal (www.cbioportal.org) databases were used to obtain data on ABAT methylation, copy number variation, and gene mutation in HCC. The data on copy number variation and gene mutation of ABAT were processed using GraphPad software (Version 8.4.3).

### Candidate miRNA Prediction

ENCORI (https://starbase.sysu.edu.cn/), which contains seven programs, including TargetScan, miRanda, PicTar, PITA, miRmap, RNA22, and microT, was utilized to predict the upstream binding miRNAs of ABAT. Only the candidate miRNAs that appeared in at least five of the programs mentioned above were selected for further analysis. miRNA data for HCC were obtained from TCGA database. The “limma” and “ggplot2” R packages were used to explore the differential expression of the candidate miRNAs of ABAT in HCC, as well as to examine the correlation between the target miRNAs and ABAT.

### Correlation Between ABAT and Glycolysis-Related Genes

Warburg has confirmed that increased glycolytic rate is an important feature of various cancers, including HCC. One cause of the “Warburg effect” is the overexpression of glycolysis-related enzymes (8). The expression of the glycolysis-related enzymes hexokinase 2 (HK2) (8), 6-phosphofructo-2-kinase/fructose 2,6-bisphosphatase 3 (PFKFB3) (9), pyruvate kinase M (PKM) (10), phosphoglycerate kinase 1 (PGK1) (11), and lactate dehydrogenase A (LDHA) (12) in HCC were evaluated using TCGA expression profile data. The correlation between ABAT and glycolysis-related enzymes was also determined. The results were displayed on a heat map.

### Relationship Between ABAT Expression and Tumor Immunity

The ESTIMATE algorithm was used to determine the immune and stromal scores, after which the R software packages “estimate” and “limma” were used to investigate the relationships of the scores with ABAT expression, based on the levels of immune infiltration. The “ggplot2” package was used to evaluate the correlations between ABAT expression and both the levels of various immune cell infiltration and the representative immune regulatory molecules in HCC. In addition, the co-expression data of ABAT and immune-related genes, including genes encoding immune activation and immunosuppressive proteins, were collected with TISIDB (http://cis.hku.hk/TISIDB/).

### Statistical Analysis

The comparisons between ABAT expression in HCC tissues and normal tissues or adjacent non-tumor tissues were drawn using t-test and Mann-Whitney test. The chi-square test was utilized to investigate the correlations between clinical characteristics and ABAT expression. The regulation of copy number variation and its relation to ABAT expression was analyzed using analysis of variance. Survival analyses in our study were performed using the Kaplan-Meier method, log-rank test, and Cox regression model. Statistical significance was set at P < 0.05. Majority of the statistical analyses were performed using R software (Version 4.0.2).

## Results

### Differential Expression of ABAT Between HCC and Normal or Adjacent Tissue Samples

**Figure 1:**
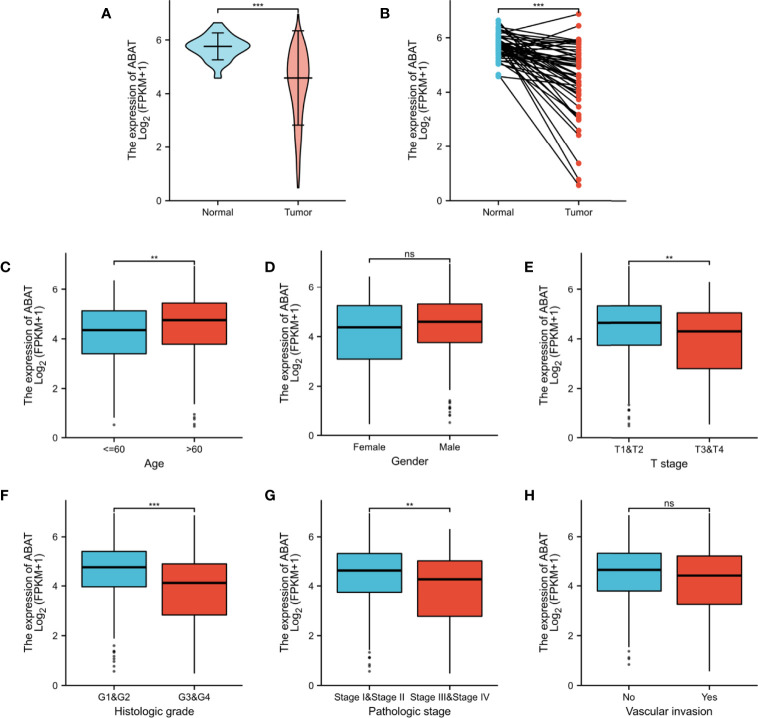
ABAT mRNA expression and its association with clinical characteristics in HCC. **(A)** Expression level of ABAT mRNA in HCC tissues and normal tissues (P < 0.001). **(B)** Expression level of ABAT mRNA in HCC tissues and adjacent tissues (P < 0.001). Association of ABAT mRNA expression with **(C)** Age, **(D)** Gender, **(E)** T stage, **(F)** Histologic grade, **(G)** Pathologic stage, **(H)** Vascular invasion. NS: P = 0.05 or higher; ^**^P < 0.01; ^***^P < 0.001.

First, TCGA database was used to investigate the transcriptional level of ABAT. The expression of ABAT mRNA was lower in HCC tissues than in normal tissues (P < 0.001) (**Figures 1A, B**). Furthermore, ABAT mRNA expression in different groups based on patient age, patient gender, T stage classification, histologic grade, pathologic stage, and vascular invasion was explored. ABAT mRNA expression in patients with HCC aged 60 years or younger was lower than that in patients older than 60 years (P = 0.004) (**Figure 1C**). The expression level of ABAT mRNA in patients with T3/T4 stage or G3/G4 grade of HCC was lower than that in patients with T1/T2 stage (P = 0.006) or G1/G2 grade (P < 0.001), respectively (**Figures 1E, F**). Patients in the late pathological stage had lower ABAT mRNA expression than that in patients in the early stage (P = 0.003) (**Figure 1G**).

**Figure 2:**
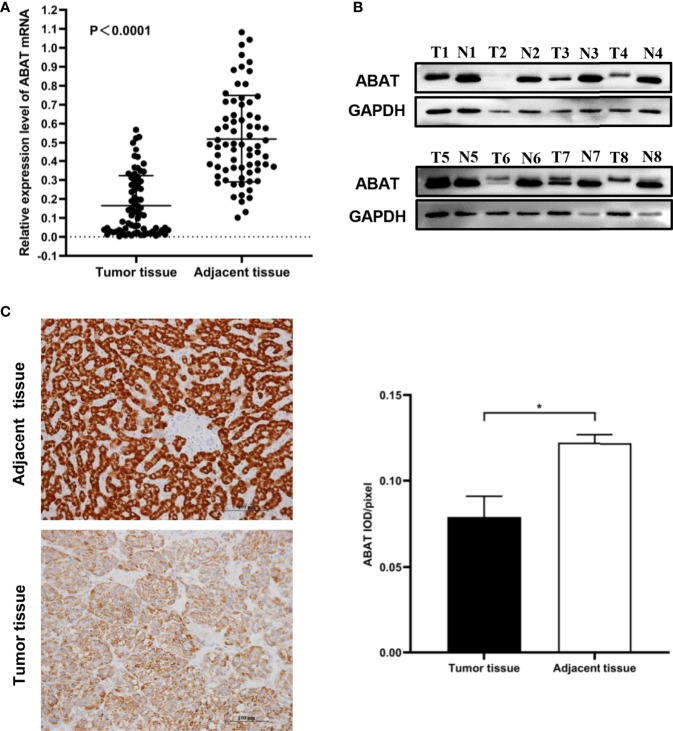
Expression of ABAT in HCC. **(A)** qRT-PCR (P < 0.0001), **(B)** Western blot, and **(C)** IHC all showed that ABAT expression was lower in HCC tissues than in adjacent tissues. ^*^P < 0.05.

We performed qRT-PCR to test ABAT expression in 71 pairs of HCC tissue samples, the result was displayed in **Figure 2A**. The results of western blotting and IHC were showed in **Figure 2B** and **Figure 2C**, respectively. Consistent with the results of bioinformatics analysis, the experimental results indicated that ABAT expression in HCC tissues was lower than that in the matched adjacent tissues.

### Low ABAT Expression in HCC Is an Independent Risk Factor

**Figure 3:**
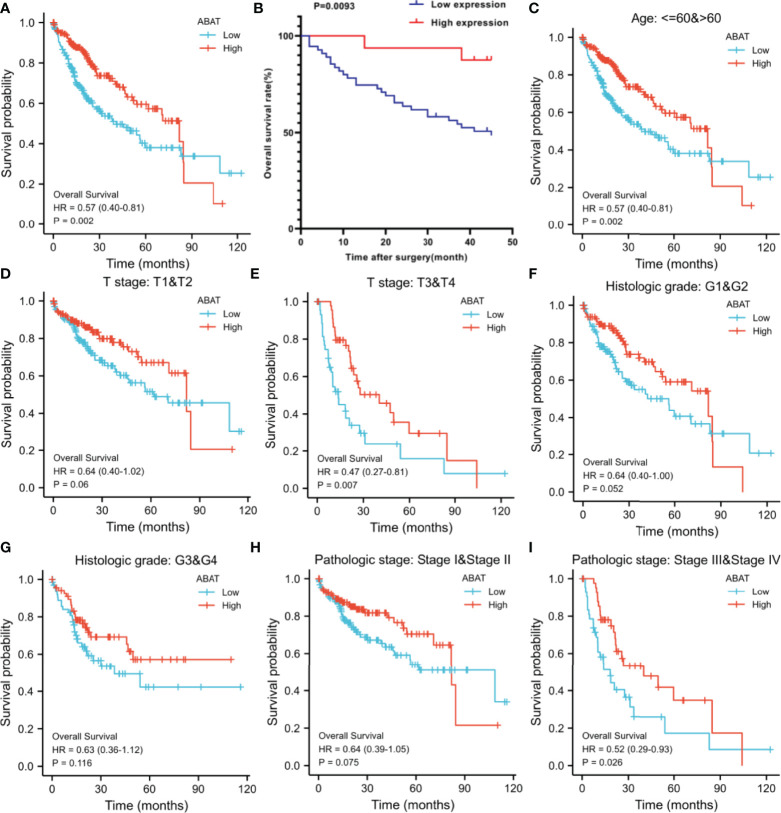
Overall survival analyses with ABAT mRNA expression. **(A)** Survival curves in HCC for all cases from TCGA. **(B)** Survival curves in HCC with the results of qRT-PCR. **(C)** Age ≤ 60 or > 60, **(D)** T stage T1/T2, **(E)** T stage T3/T4, **(F)** Histologic grade G1/G2, **(G)** Histologic grade G3/G4, **(H)** Pathologic grade I/II, **(I)** Pathologic grade III/IV.

**Figure 4:**
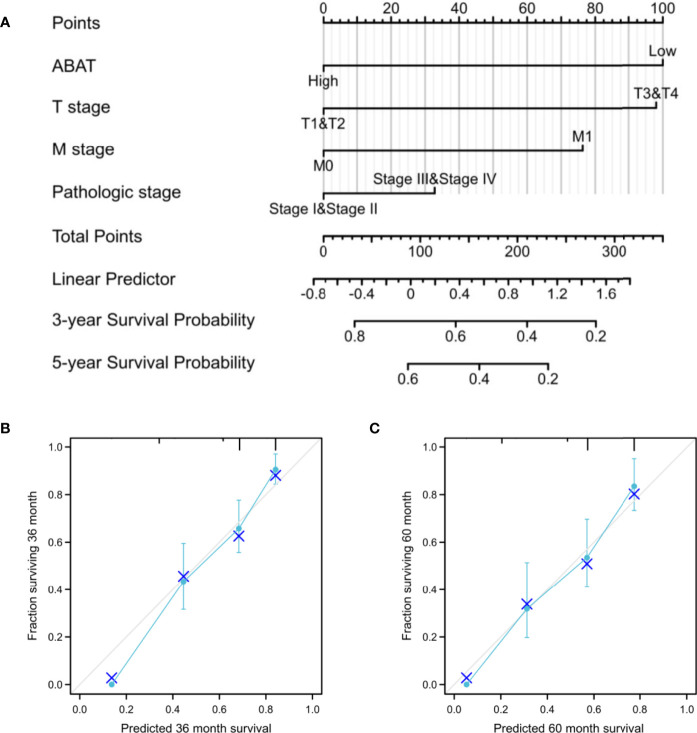
Establishment and validation of a predictive nomogram. **(A)** Nomogram to predict the probability of 3- and 5-year OS for HCC. **(B)** The calibration plot of the nomogram for outcome prediction.

Our results revealed that low ABAT mRNA expression was related to patient age (P = 0.011), T stage classification (P = 0.013), pathologic stage (P = 0.014), histologic grade (P < 0.001), and AFP level (P < 0.001) (**Table 1**). Survival analyses revealed that lower ABAT mRNA expression was correlated with poor OS in patients with HCC (P = 0.002) (**Figure 3A**), consistent with the qRT-PCR data (P = 0.0093) (**Figure 3B**). Subgroup analyses revealed that low expression of ABAT mRNA could affect OS in HCC patients aged below 60 years (P = 0.002), at T3/T4 stage (P = 0.007), and at pathological stage III/IV (P = 0.026) (**Figures 3C–I**). Univariate analysis showed that low ABAT expression, high T stage classification, advanced pathological stage, and distant metastasis were related to OS. Multivariate analysis indicated that low expression of ABAT was an independent risk factor for HCC patients (**Table 2**). We then created a nomogram to integrate ABAT as a biomarker of HCC by fitting the expression of ABAT, T stage classification, M stage, and pathological stage (**Figure 4A**). A worse prognosis factor was indicated by a higher nomogram score. The nomogram performance of ABAT was evaluated using a calibration curve. The C-index was 0.680 for ABAT. The prediction results were consistent with the observation results because the deviation line was close to the ideal curve in the calibration plot (**Figures 4B, C**).

**Table 1:** Relationship between ABAT expression and clinical characteristics.

Clinical characteristics	Levels	Low expression	High expression	P
		N (%)	N (%)	
Age	<=60	101 (27.1%)	76 (20.4%)	0.011
	>60	85 (22.8%)	111 (29.8%)	
Gender	Female	63 (16.8%)	58 (15.5%)	0.658
	Male	124 (33.2%)	129 (34.5%)	
T stage	T1	79 (21.3%)	104 (28%)	0.013
	T2	49 (13.2%)	46 (12.4%)	
	T3	52 (14%)	28 (7.5%)	
	T4	6 (1.6%)	7 (1.9%)	
N stage	N0	135 (52.3%)	119 (46.1%)	0.626
	N1	3 (1.2%)	1 (0.4%)	
M stage	M0	143 (52.6%)	125 (46%)	0.626
	M1	3 (1.1%)	1 (0.4%)	
Pathologic stage	Stage I	76 (21.7%)	97 (27.7%)	0.014
	Stage II	44 (12.6%)	43 (12.3%)	
	Stage III	55 (15.7%)	30 (8.6%)	
	Stage IV	3 (0.9%)	2 (0.6%)	
Histologic grade	G1	19 (5.1%)	36 (9.8%)	< 0.001
	G2	78 (21.1%)	100 (27.1%)	
	G3	80 (21.7%)	44 (11.9%)	
	G4	8 (2.2%)	4 (1.1%)	
AFP (ng/ml)	<=400	86 (30.7%)	129 (46.1%)	< 0.001
	>400	51 (18.2%)	14 (5%)	
Child-Pugh grade	A	107 (44.4%)	112 (46.5%)	0.909
	B	11 (4.6%)	10 (4.1%)	
	C	0 (0%)	1 (0.4%)	
Vascular invasion	No	97 (30.5%)	111 (34.9%)	0.360
	Yes	58 (18.2%)	52 (16.4%)	

**Table 2:** Univariate and multivariate Cox analysis of ABAT and other clinical pathological factors with OS in HCC patients.

Characteristics	Univariate analysis		Multivariate analysis	
	Hazard ratio (95% CI)	P value	Hazard ratio (95% CI)	P value
Age (years) (<= 60 vs > 60)	1.205 (0.850-1.708)	0.295		
Gender (Female vs Male)	0.793 (0.557-1.130)	0.200		
T stage (T1&T2 vs T3&T4)	2.598 (1.826-3.697)	<0.001	2.183 (0.296-16.098)	0.444
N stage (N0 vs N1)	2.029 (0.497-8.281)	0.324		
M stage (M0 vs M1)	4.077 (1.281-12.973)	0.017	1.827 (0.559-5.966)	0.318
Pathologic stage (I&II vs III&IV)	2.504 (1.727-3.631)	<0.001	1.299 (0.177-9.521)	0.797
Histologic grade (G1&G2 vs G3&G4)	1.091 (0.761-1.564)	0.636		
AFP (ng/ml) (<= 400 vs > 400)	1.075 (0.658-1.759)	0.772		
Child-Pugh grade (A vs B vs C)	1.595 (0.757-3.361)	0.219		
	2.138 (0.294-15.544)	0.453		
Vascular invasion (No vs Yes)	1.344 (0.887-2.035)	0.163		
ABAT (Low vs High)	0.571 (0.401-0.812)	0.002	0.451 (0.285-0.715)	<0.001

### ABAT-Related Signaling Pathways Identified *via* GSEA

**Figure 5:**
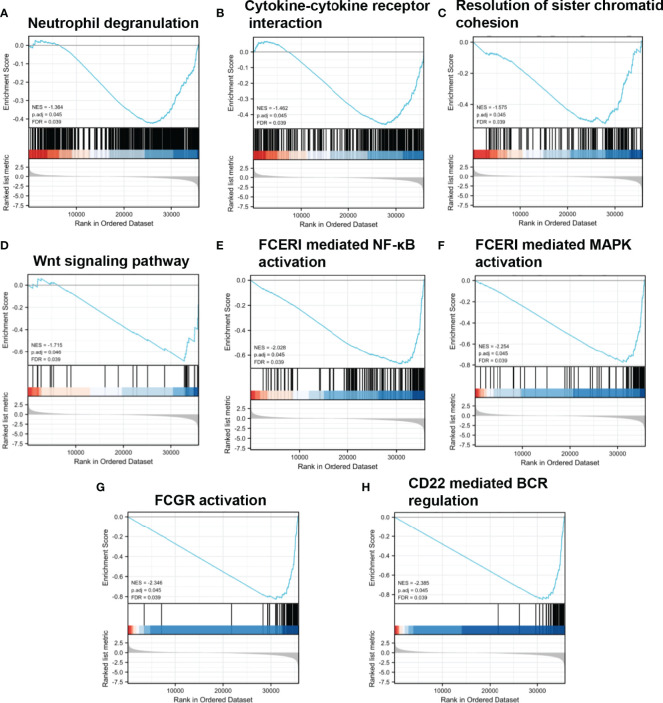
Enrichment plots from GSEA. **(A–H)** NES, normalized ES; ADJ P-val, adjusted P-value; FDR, false discovery rate.

Various signaling pathways were identified using GSEA, including “neutrophil degranulation,” “cytokine to cytokine receptor interaction,” “resolution of sister chromatid cohesion,” “WNT signaling pathway,” “FceRI-mediated NF-κB activation,” “FceRI-mediated MAPK activation,” “FCGR activation,” and “CD22-mediated BCR regulation”; these were determined using the normalized enrichment score (NES), adjusted P-value, and FDR (**Table 3**, **Figure 5**).

**Table 3:** Gene sets enriched in phenotype.

Gene set name	NES	MOMp-val	FDRq-val
REACTOME_NEUTROPHIL_DEGRANULATION	-1.364	0.001	0.039
KEGG_CYTOKINE_CYTOKINE_RECEPTOR_INTERACTION	-1.462	0.001	0.039
REACTOME_RESOLUTION_OF_SISTER_CHROMATID_COHESION	-1.575	0.001	0.039
PID_WNT_SIGNALING_PATHWAY	-1.715	0.001	0.039
REACTOME_FCERI_MEDIATED_NF_KB_ACTIVATION	-2.028	0.001	0.039
REACTOME_FCERI_MEDIATED_MAPK_ACTIVATION	-2.254	0.001	0.039
REACTOME_FCGR_ACTIVATION	-2.346	0.001	0.039
REACTOME_CD22_MEDIATED_BCR_REGULATION	-2.385	0.001	0.039

### DNA Methylation, Copy Number Variation, and Gene Mutation of ABAT

**Figure 6:**
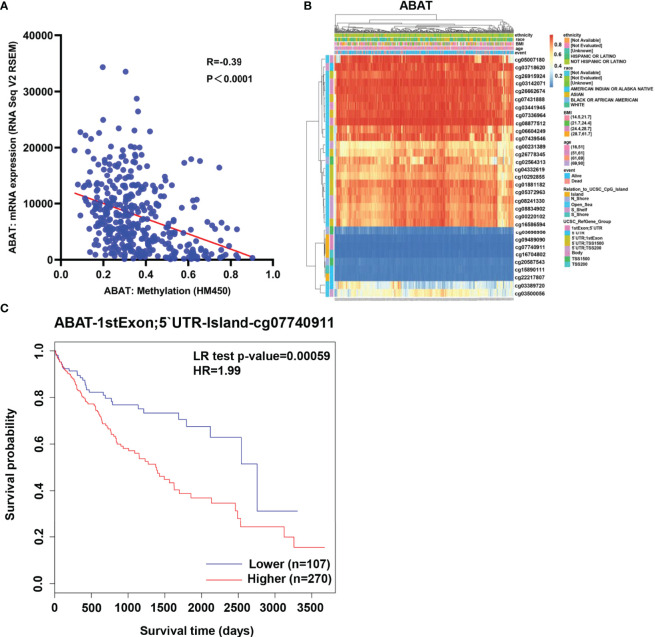
Methylation of ABAT in HCC. **(A)** The correlation between ABAT methylation and its expression. **(B)** The visualization of the methylation level and the expression of ABAT. **(C)** The survival curves for promoter methylation of ABAT.

**Figure 7:**
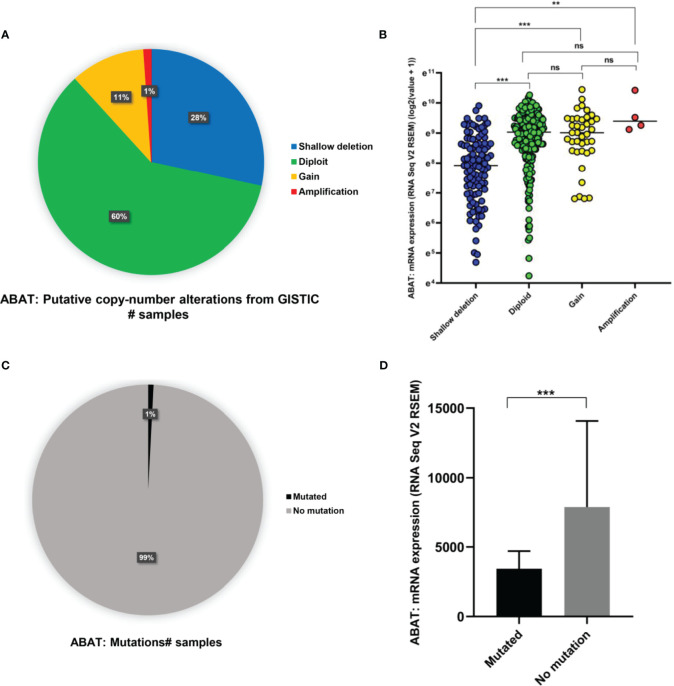
Copy number variation and gene mutation of ABAT. **(A)** The proportion of various types of copy number variations of ABAT. **(B)** The effect of copy number variation on ABAT expression. **(C)** The proportion of gene mutation and no mutation of ABAT. **(D)** The effect of gene mutation on ABAT expression. NS, P = 0.05 or higher; **P < 0.01; ***P < 0.001.

As shown in **Figure 6A**, ABAT expression had a negative correlation with its methylation level (R = -0.39, P < 0.0001). The effects of hypermethylation levels on ABAT expression and HCC prognosis were analyzed using MethSurv (**Figures 6B, C**). Hypermethylation level of ABAT correlated with a poor prognosis. The types of copy number variation include deep deletion, shallow deletion, diploid, gain, and amplification (13). From the cBioPortal data, we discovered that the proportions of ABAT gene shallow deletion, diploid, gain, and amplification were 28%, 60%, 11%, and 1%, respectively, in HCC (**Figure 7A**). **Figure 7B** shows the effect of copy number variation on ABAT gene expression. Compared to diploid, shallow deletion was associated with low expression of ABAT (P < 0.001). Gene mutation is an important factor affecting gene expression and our results revealed that the proportion of ABAT gene mutation in HCC was 1%, which was related to low ABAT expression (P < 0.001) (**Figures 7C, D**).

### miR-135a-5p Might Be an Upstream miRNA of ABAT

**Figure 8:**
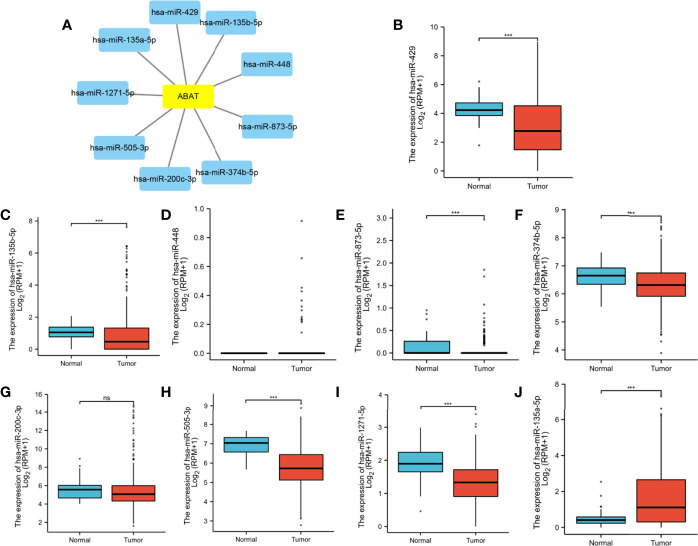
**(A)** Establishment of miRNA-ABAT regulatory network with Cytoscape software and **(B–J)** the expression of candidate upstream miRNAs of ABAT in HCC tissues and normal tissues. NS, P = 0.05 or higher; ***P < 0.001.

**Figure 9:**
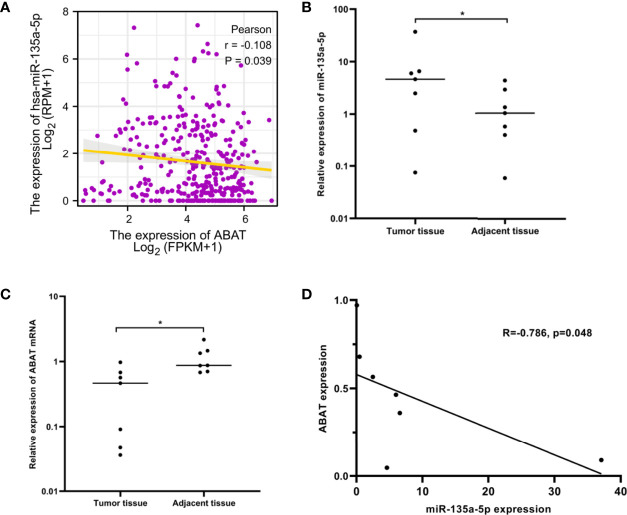
miR-135a-5p expression and its correlation with ABAT in HCC. **(A)** The correlation between miR-135a-5p and ABAT was analyzed with TCGA data. **(B)** qRT-PCR for detection of miR-135a-5p in seven paired HCC and adjacent tissues. **(C)** qRT-PCR for detection of ABAT in seven paired HCC and adjacent tissues. **(D)** The correlation between miR-135a-5p and ABAT with qRT-PCR data. ^*^P < 0.05.

Using the ENCORI database, we first identified nine upstream miRNAs that could potentially bind to ABAT. Cytoscape software was used to visualize the miRNA-ABAT regulatory network (**Figure 8A**). Based on the regulation mechanism of target genes by miRNAs, we expected that there would be a negative association between miRNAs and ABAT. Thus, the expression analysis of miRNAs in HCC was performed. Only miR-135a-5p had higher expression in HCC tissues than that in normal tissues (**Figures 8B–J**). According to TCGA data analysis, ABAT had a negative association with miR-135a-5p (**Figure 9A**).

We performed qRT-PCR to assess the expression of miR-135a-5p and ABAT mRNA in 7 pairs of HCC tissue samples. These results verified the above analysis (**Figures 9B–D**). Therefore, miR-135a-5p may be an upstream miRNA of ABAT.

### Expression of Glycolysis-Related Genes Was Negatively Correlated With ABAT Levels

**Figure 10:**
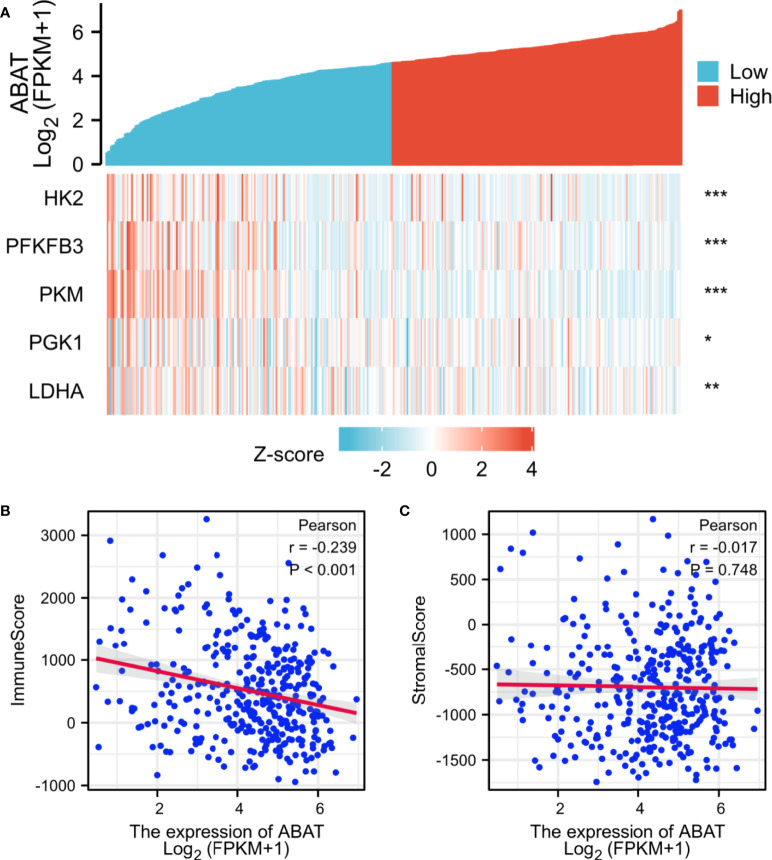
Correlations between ABAT expression and glycolysis-related genes/tumor microenvironment. **(A)** The X-axis and Y-axis represent the samples and the glycolysis-related genes, respectively. Green and red tones indicate the downregulation and upregulation of genes, respectively. **(B)** Correlation between ABAT and immune score in HCC. **(C)** Correlation between ABAT and stromal score in HCC. ^*^P < 0.05, ^**^P < 0.01, ^***^P < 0.001.

Subsequently, we explored the correlations between ABAT and glycolysis-related genes, which are vital for energy metabolism in HCC cells. The genes that we investigated included *HK2*, *PFKFB3*, *PKM*, *PGK1*, and *LDHA*. Our results revealed that the expression of glycolysis-related genes was negatively correlated with ABAT levels (**Figure 10A**).

### Correlations Between ABAT Expression and Tumor Immunization

**Figure 11:**
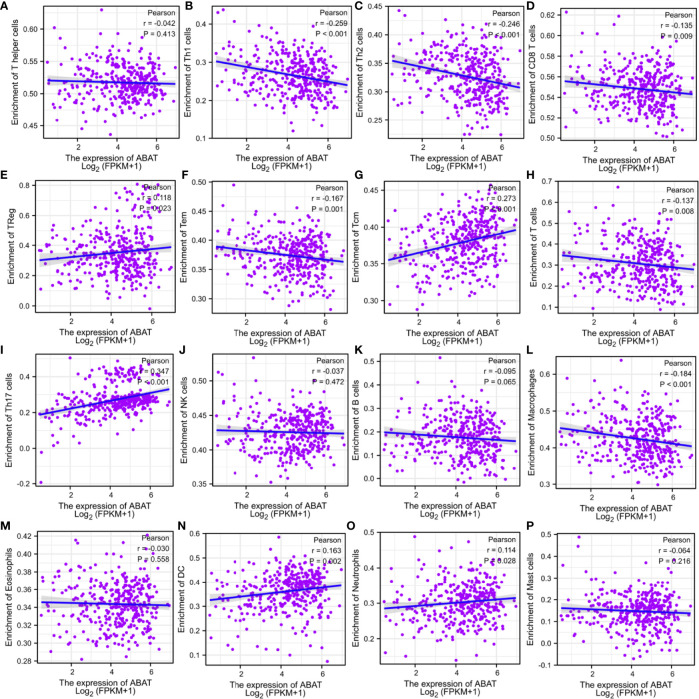
**(A–P)** Relationship between ABAT expression and tumor infiltrating immune cells.

**Figure 12:**
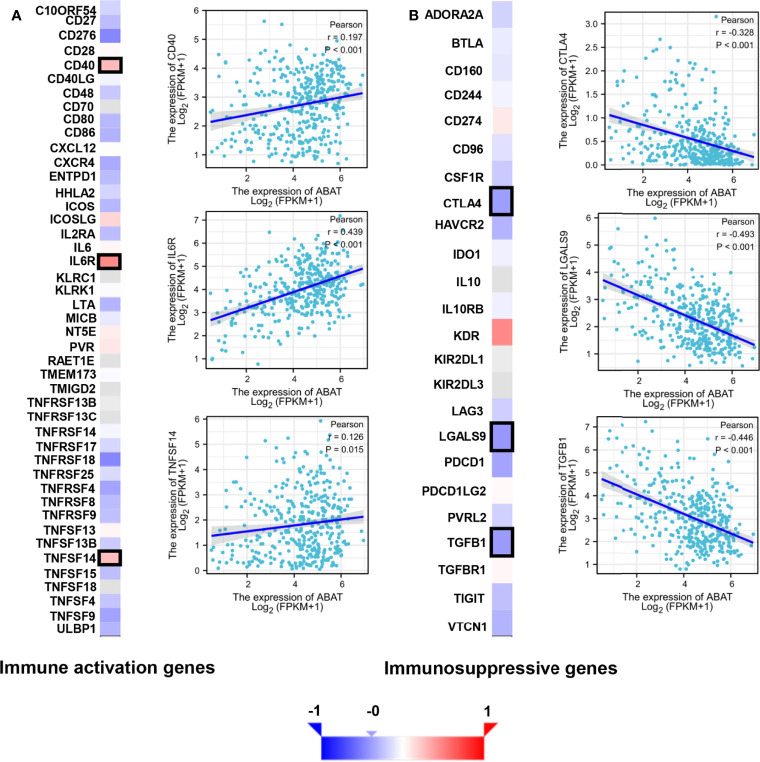
Correlation of ABAT levels with the expression of immunomodulators in HCC. **(A)** The heatmap of the correlation with immune activation genes and display of three representative molecules. **(B)** The heatmap of the correlation with immunosuppressive genes and display of three representative molecules. The representative molecules are surrounded by black frames in the heatmaps.

The tumor immune microenvironment acts as a vital factor in the occurrence and development of HCC (14). The stromal and immune cell scores in HCC were calculated using the ESTIMATE algorithm, and the correlations between ABAT and these two score types were analyzed. Immune cell score had negative correlation with ABAT expression levels (R = −0.239, P < 0.001) (**Figure 10B**), whereas stromal score was not significantly correlated (R = −0.017, P = 0.748) (**Figure 10C**). By exploring the relationships between ABAT and various immune-related cells in HCC, we determined the levels of infiltrating T cells and their different subgroups: T helper 1 (Th1) cells, T helper 2 (Th2) cells, CD8+ pan T (CD8+ T) cells, and effector memory T (Tem) cells, all of which, along with macrophages, had negative associations with ABAT expression levels. Conversely, the levels of infiltrating regulatory cells (Tregs), central memory T cells (Tcm), T helper 17 (Th17) cells, dendritic cells (DCs), and neutrophils had positive associations with ABAT expression (**Figure 11**). The correlations between ABAT expression levels and the genes encoding immune activation and immunosuppressive proteins were analyzed using TISIDB data. ABAT expression levels had positive correlations with some immune activators (**Figure 12A**) and negative correlations with almost all immunosuppressive factors (**Figure 12B**). Some representative molecules were displayed.

## Discussion

ABAT expression is downregulated in several types of cancer, including renal carcinoma (15), breast cancer (3, 16), and pancreatic cancer (17). Our study also showed that ABAT had lower expression in HCC tissues than that in normal tissues or adjacent non-tumor tissues, consistent with previous research (4). Downregulation of ABAT expression was correlated with patient age, tumor T stage classification, pathological stage, histologic grade, and AFP level of HCC and was an independent prognostic factor for HCC. Survival analysis validated that low ABAT expression was associated with poor prognosis in HCC. Similarly, loss of ABAT expression was previously documented to be strongly correlated with poor survival in basal-like breast cancer patients (3). Our results suggest that ABAT can be used as a biomarker for the determination of HCC prognosis.

Low expression of ABAT can increase the level of the substrate molecule, GABA, which can mediate the Ca^2+^-NFAT1 axis to promote tumor growth and metastasis in BLBC (3). As mentioned previously, the ABAT-GABA-Ca^2+^ signaling pathway might not be the main mechanism affecting tumor prognosis in HCC. Therefore, we explored the possible associated molecular mechanisms using GSEA, which showed that in the low-expression ABAT phenotype, pathways such as “neutrophil degranulation,” “cytokine to cytokine receptor interaction,” “resolution of sister chromatid cohesion,” “WNT signaling pathway,” “FceRI-mediated NF-κB activation,” “FceRI-mediated MAPK activation,” “FCGR activation,” and “CD22-mediated BCR regulation” were significantly differentially enriched. Among them, the tumor progression-related pathways were “resolution of sister chromatid cohesion” and “WNT signaling pathway.” The WNT/β-catenin pathway can regulate embryonic development, cellular proliferation, and differentiation, and its aberrant expression is related to tumor stem cell maintenance, tumor progression, and drug resistance in HCC (18, 19). Additionally, the signaling pathways implicated in immune and inflammatory responses included “neutrophil degranulation,” “cytokine to cytokine receptor interaction,” “FceRI-mediated NF-κB activation,” “FceRI-mediated MAPK activation,” “FCGR activation,” and “CD22-mediated BCR regulation.” Inflammatory cytokines can drive the progression of HCC. Furthermore, although adaptive immunity helps to eradicate early HCC, immune cells can also provoke HCC development. Therefore, the role of local immunity in HCC development is a complicated topic (20). Together, our results revealed that ABAT is involved in tumor progression and immune-related pathways.

We further investigated the mechanisms involved in low ABAT expression in HCC. ABAT hypermethylation, shallow deletion, and gene mutation were all associated with low ABAT expression in HCC, and patients with hypermethylations had a poor prognosis. DNA methylation, which regulates gene expression, is an epigenetic mechanism. Zhao et al. reported that ABAT expression was substantially downregulated in patients with myelodysplastic syndrome (MDS) compared with that in controls and that ABAT methylation level increased in MDS patients. ABAT methylation can affect MDS progression and is considered a sign of poor prognosis in hematological tumors (21). MiRNAs, a type of small endogenous RNAs, can regulate gene expression by targeting gene mRNAs (22). Bioinformatics analysis suggested that miR-135a-5p might be an upstream miRNA of ABAT, and our experimental results verified this. Indeed, miR-135a-5p can affect tumor progression by targeting correlated genes (23–25). Van Renne et al. found that miR-135a-5p upregulation in hepatitis C virus (HCV)-infected livers could drive HCV-associated hepatocarcinogenesis by targeting protein tyrosine phosphatase receptor delta (26). Yao et al. demonstrated that miR-135a-5p expression was upregulated in HCC and was negatively related to Krüppel-like Factor-4 expression, which promoted the proliferation and metastasis of HCC (27). Furthermore, miR-183-5p is the upstream miRNA of ABAT and can regulate cell functions in liver cancer (4), although this finding is inconsistent with our results. This may be because each miRNA can regulate the expression of multiple target genes, and each target gene can also be regulated by multiple miRNAs simultaneously (28).

Warburg first described the enhanced conversion of glucose into lactate in malignant tumors, even in an aerobic environment (29). This phenomenon is known as aerobic glycolysis or the Warburg effect. Aerobic glycolysis was first discovered in patients with HCC. It acts as a regulatory factor in the proliferation, angiogenesis, metastasis, and drug resistance of HCC (30). Our results determined that the expression of the glycolysis-related genes *HK2*, *PFKFB3*, *PKM*, *PGK1*, and *LDHA* were negatively correlated with ABAT expression levels. Sun et al. revealed that the androgen receptor could upregulate *HK2* expression, which induces glycolysis *via* the PKA/CREB signaling pathway, promoting the progression of HCC (8). Long et al. found that PFKFB3 expression was significantly upregulated in both HCC tissues and cell lines and was correlated with sorafenib resistance in HCC patients (9). The two isoforms of PKM, PKM1 and PKM2, are produced by the splicing of PKM. The long non-coding RNA LNCAROD could induce the upregulation of PKM2, eventually increasing cancer cell aerobic glycolysis, which is related to poor prognosis in HCC (10). PGK1 is an important glycolysis-related enzyme. Xie et al. observed that PGK1 was overexpressed in HCC tissues and that it could enhance the Warburg effect by inducing metabolic reprogramming; also, high PGK1 expression was correlated with bad prognosis in HCC patients (11). LDHA plays a vital role in non-neoplastic and neoplastic cells through the catalysis of pyruvate into lactate. Sheng et al. found that LDHA overexpression was correlated with a low apoptosis rate and high metastasis of HCC cells (12). Altogether, ABAT may be involved in the reprogramming of HCC energy metabolism.

Apart from cancer cells, the tumor microenvironment includes various components, including immune-related cells, stromal cells, fibroblasts, extracellular matrix, and signaling molecules, and is related to tumor growth, metastasis, and prognosis (31). Our results showed that most signaling pathways, as determined *via* GSEA, were implicated in the immune and inflammatory responses in HCC. Similarly, the ESTIMATE scores demonstrated that immune cell score was negatively correlated with ABAT expression levels. The ssGSEA revealed that the levels of infiltrating T cells and their different subgroups (Th1, Th2, CD8+ T, and Tem cells, as well as macrophages) were negatively associated with the ABAT expression levels. In contrast, ABAT expression levels were significantly associated with the infiltration levels of Treg, Tcm, Th17, and DC cells, as well as neutrophils. These results suggest that immune infiltration may be an aspect of ABAT-mediated carcinogenesis in HCC. Additionally, sufficient infiltration of immune cells and abundant expression of immune checkpoint molecules are all needed for the efficacy of immunotherapy (32). Our study validated the co-expression of ABAT with immune activation and immunosuppressive proteins. ABAT was negatively correlated with almost all immunosuppressive factors, such as CTLA4, LGALS9, and TGFB1 in HCC, indicating that ABAT expression might be associated with the efficacy of immunotherapy in HCC.

This research also has certain limitations. First, the quality of the data from public databases could not be assessed. Second, the specific mechanism of ABAT in HCC could not be determined and needs further exploration.

In conclusion, our study confirmed that ABAT expression was downregulated in HCC. Low ABAT expression was associated with poor prognosis and was an independent risk factor in HCC patients. ABAT might promote tumor progression through a variety of signaling pathways, including the WNT signaling pathway, FceRI-mediated NF-κB activation, and FceRI-mediated MAPK activation. The mechanism of low ABAT expression might involve ABAT hypermethylation, shallow deletion, and gene mutation, and miR-135a-5p may be an upstream miRNA of ABAT. Furthermore, ABAT might be involved in energy metabolism reprogramming, immune cell infiltration, and the expression of immunosuppressive factors.

## Data Availability Statement

The datasets presented in this study can be found in online repositories. The names of the repository/repositories and accession number(s) can be found in the article/supplementary material.

## Ethics Statement

The studies involving human participants were reviewed and approved by the Ethics Committee of the Fifth Medical Center of Chinese PLA General Hospital. Written informed consent was obtained from the individual(s) for the publication of any potentially identifiable images or data included in this article.

## Author Contributions

SZ and YLu designed this research. XG, XJ, JX, and JL collected the samples and data. XG and XJ conducted qRT-PCR, IHC, and western blotting and analyzed the data. XG wrote the first draft. SZ, YLu, MX, and YLi supervised the research and revised the manuscript. All authors contributed to the article and approved the submitted version.

## Funding

This study was supported by grants from Natural Science Foundation of Beijing Municipality (7212099), National Natural Science Foundation of China (81902495), Medical Big Data and AI R & D Project of General Hospital (2019MBD-025), and Science Technology and Innovation Committee of Shenzhen Municipality (KCXFZ202002011006448).

## Conflict of Interest

The authors declare that the research was conducted in the absence of any commercial or financial relationships that could be construed as a potential conflict of interest.

## Publisher’s Note

All claims expressed in this article are solely those of the authors and do not necessarily represent those of their affiliated organizations, or those of the publisher, the editors and the reviewers. Any product that may be evaluated in this article, or claim that may be made by its manufacturer, is not guaranteed or endorsed by the publisher.
